# Behavioral Correlates of Primates Conservation Status: Intrinsic Vulnerability to Anthropogenic Threats

**DOI:** 10.1371/journal.pone.0135585

**Published:** 2015-10-07

**Authors:** Amélie Christelle Lootvoet, Justine Philippon, Carmen Bessa-Gomes

**Affiliations:** 1 Laboratory “Ecologie, Systématique, Evolution”, UMR 8079, University Paris-Sud, Bâtiment 362, Orsay, France; 2 Laboratory “Ecologie, Systématique, Evolution”, UMR 8079, CNRS, Bâtiment 362, Orsay, France; 3 Laboratory “Ecologie, Systématique, Evolution”, UMR 8079, AgroParisTech, Bâtiment 362, Orsay, France; Federal University of Goiás, BRAZIL

## Abstract

Behavioral traits are likely to influence species vulnerability to anthropogenic threats and in consequence, their risk of extinction. Several studies have addressed this question and have highlighted a correlation between reproductive strategies and different viability proxies, such as introduction success and local extinction risk. Yet, very few studies have investigated the effective impact of social behaviour, and evidence regarding global extinction risk remains scant. Here we examined the effects of three main behavioral factors: the group size, the social and reproductive system, and the strength of sexual selection on global extinction risk. Using Primates as biological model, we performed comparative analysis on 93 species. The conservation status as described by the IUCN Red List was considered as a proxy for extinction risk. In addition, we added previously identified intrinsic factors of vulnerability to extinction, and a measure of the strength of the human impact for each species, described by the human footprint. Our analysis highlighted a significant effect of two of the three studied behavioral traits, group size and social and reproductive system. Extinction risk is negatively correlated with mean group size, which may be due to an Allee effect resulting from the difficulties for solitary and monogamous species to find a partner at low densities. Our results also indicate that species with a flexible mating system are less vulnerable. Taking into account these behavioral variables is thus of high importance when establishing conservation plans, particularly when assessing species relative vulnerability.

## Introduction

There is increasing evidence that we may be facing a sixth mass extinction in the near future [1,2]. Species extinction rates are very high, and the survival of many other species is threatened [3]. The causes of population decline and present vulnerability to extinction are mainly anthropogenic, and can be classified into six categories including habitat degradation, overexploitation, invasion of non-native species, climate change, pollution, and spread of disease [2,4,5]. These threats constitute the extrinsic factors of vulnerability. Because they are not distributed homogeneously across the world, the extrinsic factors of vulnerability do not concern all the species. The degree of exposure to extrinsic factors is very likely the primary factor leading to species extinction risk. Nevertheless, species living in the same area, and experiencing the same anthropogenic pressure are not necessarily at the same risk of extinction and will not share the same conservation status as measured by the IUCN Red List [6]. This is because several species-specific characteristics can modulate species vulnerability to extinction threats and to extinction. These characteristics are known as “intrinsic factors of vulnerability” and include life history traits and ecological characteristics. Two life history traits often identified as intrinsic factors of vulnerability are gestation length and body mass. The impact of gestation length on species vulnerability is well documented: species with longer gestation time are more prone to extinction because of their longer recovery time [7–9]. On the contrary, the impact of body mass on extinction risk is more controversial. While many studies highlighted that the higher the body mass the more species are at risk of extinction [7,10–13], several other studies did not find any body mass effect [14–16]. The higher vulnerability of large-bodied species is usually explained by lower densities of large-bodied species [17], higher requirements (for diet and habitat [18,19]), and longer recovery times (due to a correlation between gestation length and body mass [8]). Concerning the ecological variables impacting the extinction risk, geographic range, population density, and trophic level may affect species vulnerability to extinction risk [8,20]. Species with small geographic range and low population density have less ability to recover from any potential perturbation, and are thus more prone to extinction [8]. High trophic level species would be more at risk because of their dependence on lowered-chain species to survive [8].

While many studies have investigated the impact of life history and ecological characteristics on the extinction risk, very few have focused on the potential influence of behavioral variables. Yet, the idea that behavior could have an important role in the extinction risks of many species is quite old, and has often been raised [21–24]. Social system and sexual selection are among the most cited behaviour variables to potentially affect extinction risk. Social system may impact species vulnerability through group size, social organisation or mating system. In group-living species, several behavioral components are likely to require a threshold number of conspecifics, like raising youngs [25], reducing predation risk (either by dilution [26,27], or by active watching [28,29]), or foraging [30,31]. Group living as long been hypothesised as a possible vulnerability factors [25], but evidence remains scant. Nevertheless, when examining mammals extinction risk as evaluated bu the UICN, Davidson and colleagues [32] highlighted a negative correlation between group size and extinction risk, for species with relatively slow life cycle and restricted distribution range (for more details, see Davidson and colleagues [32]). Species extinction risk may also depend on social organisation, since solitary species are less likely to find a mate when population density becomes too low [32]. Another social system component proposed to contribute to extinction risk is the mating system, but conclusions about its impact are diverse. A theoretical study regarding the effect of the mating system on extinction risk showed that all mating systems are likely to experience a strong Allee effect when the operational sex ratio (OSR) is balanced [33]. Hence, the demographic structure is paramount to understand the impact of mating systems. Monogamy may be more likely to approach a balanced OSR than polygyny, but anthropogenic disturbance such as selective hunting may render polygynous species more extinction prone [33]. Moreover, polygyny has the potential to result in higher demographic variance [34]. One study in birds found no effect of mating system on the extinction risk [33], whereas a study based on Mammals assessed that monogamous and polygynous species living in small groups were more at risk [35]. Several studies have also assumed an influence of sexual selection in species extinction risk [36,37], but its real impact remains hard to demonstrate. A comparative analyse using an extensive database on bird introductions concluded that sexual selection was negatively correlated to introduction success [38]. This result was supported by ulterior analysis of local extinction rate [39] and conservation status of North American birds [40], but not by the comparative analysis of the conservation status of European birds and Mammals [41]. Despite non-concordant results, group size, socio-reproductive system and sexual dimorphism are often mentioned as potentially intrinsic factors of vulnerability in studies on the causes of extinction risks.

The main aim of this study is to examine the role of three major behavioral components in the extinction risk in Primate species: group size, social and mating system, and sexual dimorphism. The choice of Primates as study model was made for three reasons. First, they present a large variety of social systems [42] and a wide range of sexual selection intensity [43]. Second, as Primates constitute one of the best-studied taxa, the behavioral data available is numerous compared to many other species groups. Finally, more than 50% of the Primate species are currently threatened by extinction [6], which renders the study of their vulnerability particularly relevant. Because Primates have been the objects of previous studies concerning intrinsic vulnerability factors, we included in our study previously identified factors relevant to the explanation of extinction risk, in order to prevent bias [43,44]. In addition, as Primates are also submitted to anthropogenic pressures, it was important to take it into account in the analysis. If not, we may have lost an important part of the variance explaining the extinction risk. In other words, because we suspected the extrinsic factors of vulnerability to be responsible in a high proportion for species conservation status, we had to include a proxy of human impact in our models, the human footprint index [45].

## Materials and Methods

### Data collection

#### Conservation status

We chose the IUCN status from the IUCN Red List (2013) as a proxy of extinction risk (downloaded from the IUCN Red List website, http://www.iucnredlist.org/). The IUCN status of threat downloaded from the IUCN Red List are part of a qualitative nominal variable with five modalities: “Least Concern”, “Near Threatened”, “Vulnerable”, “Endangered” and “Critical” (LC, NT, VU, EN and CR). For our analysis we transformed the IUCN status into an ordinal variable (0, 1, 2, 3, 4, respectively). This type of transformation is commonly used in other studies using the IUCN status of threat as a proxy of species extinction risk [8,16,35,44].

#### Extrinsic factors of vulnerability

We chose the human footprint index (version 2: 1995–2004) as an indicator of the strength of the extrinsic factors of vulnerability. The human footprint index measures the impact of human societies at the surface of Earth [45]. It combines four variables: human population density, land transformation, electrical power infrastructures and accessibility (for more details, see [45]). The use of the human footprint index as a proxy of human impact on terrestrial ecosystems presents at least three advantages. First, the data contained in this index provide good information of the main threats that affect Primate species. Primates are mainly threatened by habitat degradation and hunting [46], which we believe should be well described by the human population density/land transformation variables of human footprint and the human population density/accessibility variables, respectively. The link between land transformation and habitat degradation seems obvious, and several studies have found a correlation between human density and hunting intensity [47,48] (however it might not be the case at a very local scale [49]), or for habitat loss [50,51]. Additionally, accessibility to Primates by roads may lead to an increase of hunting intensity [46]. Second, the human footprint index is available for the distribution range of all Primate species. Finally, to our knowledge, the influence of human footprint on Primate extinction risk has never been studied at a global scale, despite its potential important effect on Primates population dynamics.

In this study, we focused on the mean human footprint in species distribution range. Data on the worldwide human footprint was downloaded from the NASA SocioEconomic Data and Applications Center (SEDAC) website (http://sedac.ciesin.columbia.edu/). Polygons representing Primate species distribution range were downloaded from the IUCN website, as ESRI shapefiles format. Polygons result from the liaison of occurrence points of the species, expended to take into account species habitat preference, where unsuitable habitats are removed. Thus, polygons provide the information that species probably occurs within it, but do not mean that it is distributed equally or occurs everywhere within that polygon.

#### Intrinsic factors of vulnerability

We examined three behavioral variables: social and mating system, average group size, and sexual dimorphism. The social organisation and the mating system were combined into a single qualitative variable because both are strongly correlated (species living in pairs are always monogamous, species living in group are either polygynous or promiscuous, and solitary species are polygynous). This variable will be referred as the Social and Mating System. The modalities were defined as follow: solitary, monogamous, polygynous, promiscuous, and polygynous-promiscuous species (i.e., species for which the mating system varies among groups). Those categories are adapted from Eisenberg and colleagues work on Primate social systems [52]. The average group size was chosen in order to assess species vulnerability to threats according to their group size. As a surrogate of sexual selection, we chose the weight sexual dimorphism, which is often used to indicate the strength of sexual selection [53].

Species life history traits included in our analysis were gestation length and body mass. As ecological variables, we selected the percentage of frugivory (i.e. the percentage of fruits in the diet of a species) and the mean home range (i.e. the mean living area of an individual/pair/group). Three climatic variables indicating the ecological flexibility of a species were also added in the models: the annual temperature range, the mean annual precipitations, and the annual precipitation range. Data concerning these climatic variables were collated on the NASA website. The annual temperature and precipitation ranges were calculated as the difference between the mean highest annual temperatures/precipitations over the distribution area with the mean lowest annual temperatures/precipitations among this same area. The mean annual precipitations were measured as the mean of all mean annual precipitation pixels among the distribution area.

Data were collated from peer-reviewed articles and online scientific databases. In order to minimise the bias due to multiple sources of information, data for each variable was collected from comparative analysis published in scientific articles and scientific databases (All the world’s Primates [54]; panTHERIA [55]. Final dataset comprised 93 Primate species for each we disposed of information on all factors.

### Data transformations

Prior to the analysis, some explicative variables were transformed to ensure a normal distribution. A log-transformation was applied to the explanatory variables that did not have zero values (female body mass, male body mass, average group size, home range). Because the gestation length and the female body mass were highly correlated (Spearman’s correlation test: rho = 0.73, p-v = 2.1e-15), we performed a linear regression between those two variables. Instead of the log(body mass), we used the residuals of this regression in our models, which correspond to the part of the female body mass that is not explained by the gestation. The same method was used to obtain a variable indicating the weight sexual dimorphism (i.e. the mean weight difference between female body mass and male body mass): we took the residuals corresponding to the part of the male body mass not explained by the female body mass [53].

### Tests of phylogenetic signal

**Fig 1:**
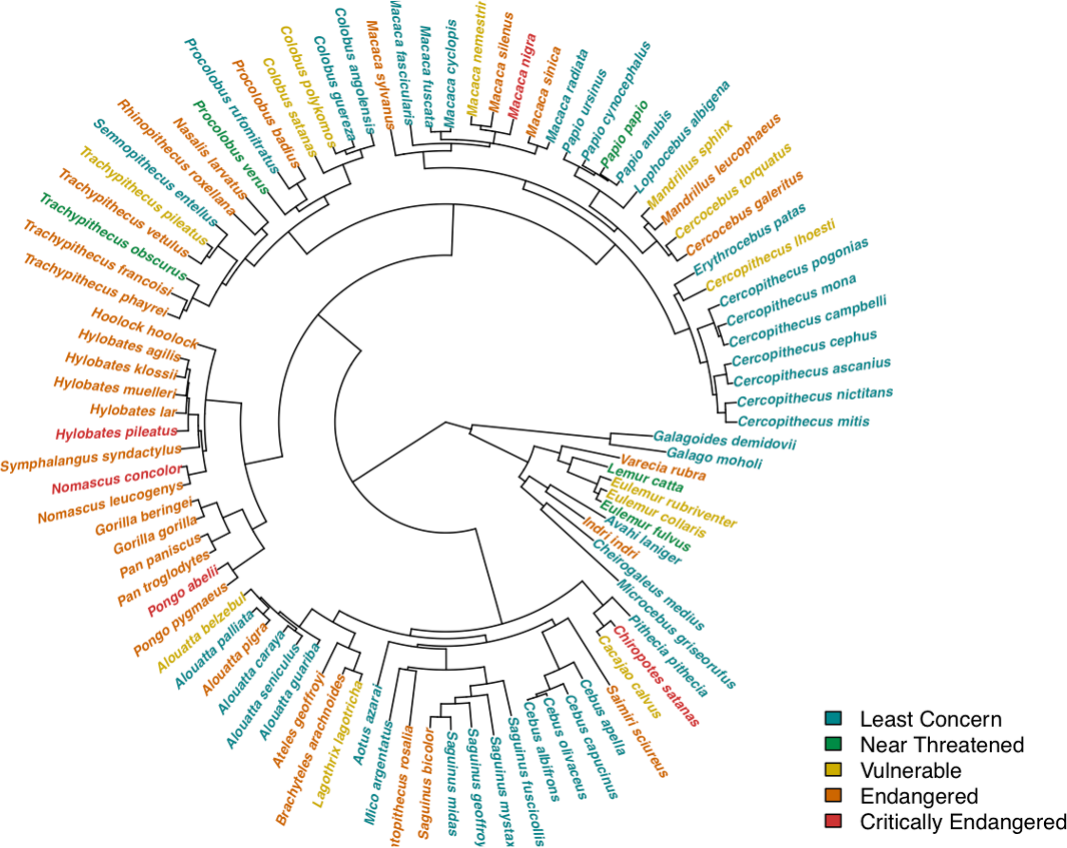
The phylogenetic inertia of the conservation status in Primate species. Species names are written in different colours according to their conservation status.

A common issue in comparative analysis is that compared species are not phylogenetically independent. This can introduce an error in the analysis when the response variable is not explained by the explicative variables, but by species proximity in the phylogeny. To prevent this error, it is necessary to test for the presence of a significant phylogenetic signal in the response variable, before starting the analysis. The presence of a phylogenetic signal in the status of threat was tested by measuring Pagel’s lambda [56], one of the most used indicator of phylogenetic signal. Even if this metric was originally designed for continuous variables, the function FitDiscrete in package Geiger in R allows its calculation for discrete traits [57,58]. We found a lambda of 0.78, indicating a positive phylogenetic signal (i.e. close related species are more probable to have the same status than species taken randomly in the phylogeny, see also Fig 1).

### Statistical models

As we measured a significant positive phylogenetic signal in the response variable (i.e. transformed extinction risk), we performed models correcting for the phylogenetic inertia [59–61]. We chose to use “Phylogenetic generalized Least Squares” (PGLSs), one of the most used methods of correction for phylogenetic inertia. PGLSs are particularly appropriate in our case, since they allow the consideration of categorical variable, which is the case of the conservation status we used as response variable [62–64]. The lambda value of 0.78 was incorporated into the models to correct for the phylogenetic non-independence of the conservation status. We began by a PGLS model containing all the explicative variables, and we adopted a top-down approach, removing at each step the less significant variable (among the insignificant ones). For each model performed, we measured the AICc (a measure of AIC for small datasets) and the ΔAICc (i.e. the difference between the AICc of the model, and the smallest AICc of all models). All models with ΔAICc<2 can be considered as the best models (Burnham & Anderson, 2002). Statistical analyses and SIG manipulations were conducted using R software (Development-Core Team 2011, packages geiger, maptools, raster and rgdal).

## Results

Several models can be considered as the best model, since five models had a ΔAICc < 2. The five models were relatively consistent, and most variables were significant in all the best models (Table 1 and S1 File). All models highlighted a significant impact of the extrinsic factor of vulnerability to extinction risk, with higher vulnerabilities of species experiencing high mean human footprint values in their distribution areas. Many intrinsic factors were also consistently present in all models and significant. Ecological factors such as the ecological flexibility, home range size and percentage of frugivory were significant in each of the 5 models, with higher extinction risk for low flexible, large home range and highly frugivorous species, respectively. On the contrary, life history traits were not present in all models, and even when present they where not consistently significant.

**Table 1:** Best explanatory models according to the AICc (ΔAICc ≤ 2 in relation the smaller AICc).

		AICc	288.20	288.93	289.44	289.44	289.77
		AICc	-	0.73	1.24	1.24	1.57
Extrinsinc factors	Mean human footprint		+++	+++	+++	+++	+++
Intrinsic Factors: *Ecology*	Annual range of precipitations		−−−	−−−	−−−	−−−	−−−
	Mean temperature range		−−−	−−	−−	−−−	−−−
	Frugivory		+++	+++	+++	+++	+++
	Home range (log)		+++	+++	+++	+++	+++
*Life history*	Gestation		+++	+	+++		+++
	Female body mass (res)		+		+		+
*Behaviour*	Mean group size (log)		−−	−−−	−−	−−	−−−
	Socio-reproductive system:						
	polygynous and promiscuous		−				−
	polygynous		+				+
	promiscuous		+				+
	solitary		−				−
	Sexual dimorphism						−

Signs indicate the sense of the effect of the variables included in the model. The number of signs inform on the significance level of the estimates (***: p ≤ 0.05, **: 0.05 < p ≤ 0.1, and * p > 0.1).

Concerning the behavioural traits, all models pointed out to a significant impact of the mean group size in the vulnerability to extinction. According to our results, species with a higher mean group size presented a lower extinction risk. Interestingly, two of the models selected incorporated the social and mating system as an explicative variable, but this variable was not significant. Finally, the model with the lowest AICc among our 5 best models also included sexual dimorphism, even though this variable was not significant.

## Discussion

This study is one of the first to highlight the existence of a behavioural dimension in species’ intrinsic vulnerability factors, and this while controlling for the intensity of extrinsic factors of vulnerability. More precisely, the average group size of Primate species has an impact on the conservation status, with species living in small groups being more vulnerable to extinction than those living in large groups. This result is consistent with the pattern highlighted by Davidson and colleagues [32], when considering mammals extinction risk. This pattern could be explained by the occurrence of an Allee effect in species living in small groups, since a well-known source of Allee effect is the difficulty to find a partner [25,65], which is supposed to be more difficult for solitary and monogamous species, (for monogamous versus polygynous species [66]). Although we did not find a significant effect of the “social and mating system” variable in this study, the model with the smallest AICc was the one including this variable in the explicative parameters. In other words, species conservation status is better explained when incorporating the “social and mating system” variable in the model, despite the absence of a significant effect. Although hardly interpretable without supplementary investigations, this might be due to a hidden real effect of the social and mating system variable. Indeed, this variable is a qualitative variable with 5 modalities, which requires more statistical power than the other variables, and it is possible we did not use enough data to detect effects in this variable

We also identified various additional intrinsic factors of vulnerability, and that included previously identified ecological and life-history traits [8,35,67]. As intrinsic factors of vulnerability other than behaviour, we found in every model a negative correlation between ecological flexibility and extinction risk (two variables indicating ecological flexibility were significant in each case). This indicates that more ecologically flexible species are more tolerant to environmental changes and disturbances, and able to better adapt to disturbed habitats. This is consistent with previous studies in Primates where increased vulnerability to logging is related to low ecological flexibility [68]. Two other ecological variables were significant in all models: species with large home range and high degree of frugivory are more at risk. The effect of home range may be due to higher ecological requirements of species with large home range to survive. If these species necessitate a high quantity of food or a wider range of habitats, they may be less tolerant toward anthropogenic perturbations. Additionally, the impact of home range size found here might also be a masked effect of the mean group size, since a large home range is quite correlated with a high mean group size (Pearson’s correlation test, cor = 0.67). The higher vulnerability of highly frugivore species is concordant with most of previous studies, and could be attributed to frugivore species dependence on scarce and patchy food resource [66], which makes them more vulnerable to habitat disturbance ([69], cited by [18]) and possibly to hunting [68].

In some of the 5 selected models, gestation length had a significant impact on the extinction risk, while the part of female body mass unexplained by gestation length had a trend to be higher with higher extinction risks. The higher vulnerability to extinction of species with higher gestation length is well-known and often highlighted in field studies (in Birds [70], in Carnivores and Primates [8], and in Mammals [44]). This is due to a longer recovery rate of species with long gestation length ([71] cited by [8]). In our study, we were able to disentangle the impact of gestation length and female body mass, which are highly correlated, by a linear regression approach. Because of this transformation, we can affirm the trend obtained for the body mass is a proper effect of the body mass and not a masked effect of gestation length (due to the high correlation of those two variables). Thus, we cannot input the increase of extinction risk with body mass to the lower recovery time of large body mass species, because the recovery time depends primarily on gestation length. The increase of extinction risk with body mass could be due to higher requirements of large body mass species (for example, large area requirements, greater food intake or high habitat specificity [72,73], or to an inverse correlation between body mass and population size [17]. An alternative explanation, less common but potentially relevant in Primates, would be that species with higher body mass are more targeted by hunters [46].

Our results confirm the low tolerance of Primate species to anthropogenic pressures [46,68], since species with higher mean human footprints were significantly more at risk of extinction, whatever the model. In our study, we only focused on the mean human footprint, which provides good bases to investigate the impact of anthropogenic pressures on the extinction risk. However, the human footprint is never homogeneous in species distribution areas. In reality, some regions among the distribution area of a species can be highly impacted by human activities, whereas some others are much more preserved [6], with the intensity of the heterogeneity depending on species. Thus, species that can take refuge in low human footprint regions in their distribution area may be less vulnerable to extinction than species that cannot benefit of from refuge zones, for a similar mean human footprint. A future important point to investigate will be to take into account the heterogeneity of the human footprint.

Another point to consider with caution is the use of IUCN conservation status as proxies for extinction risk. The conversion of IUCN status into a quantitative variable ranging from 0 to 4 could induce an error because it leads to a quantification of the extinction risk that did not exist in the raw IUCN status. Thus, it is at high interest that future studies investigate the way we could transform IUCN status into a variable that does not introduce errors.

Our work is one of the first to highlight the impact of behavior in the extinction risk in Primates, by disentangling the effects of different factors of vulnerability, both extrinsic and intrinsic. By doing so, we provide new elements to answer the old and hard-to-investigate question of the impact of behaviour in the extinction risk. In addition, identifying the factors responsible for the extinction of Primates, by which more than 50% of species are threatened of extinction, is critical to establish successful conservation plans. Indeed, previous works [74,75] highlighted the importance of identifying priority areas for conservation and integrating the information on intrinsic factors of vulnerability when establishing conservation plans is particularly relevant. Thus, knowing some behavioural aspects render species more prone to extinction can lead to specific measures in conservation plans, to counter the negative impacts of some behaviors. By doing so, the preservation of populations threatened by extinction should be more efficient.

## Supporting Information

S1 File The results of statistical analysis PGLS models. **Models presented here are the one with the smallest AICc, and other models which have** Δ**AICc <2.**
(PDF)
