# Supplementary material for: Behavioral Correlates of Primates Conservation Status: Intrinsic Vulnerability to Anthropogenic Threats
Source: PLoS One. 2015 Oct 7;10(10):e0135585. doi: 10.1371/journal.pone.0135585 (PMC4596868; doi:10.1371/journal.pone.0135585)
Supplement: S1 File — Models presented here are the one with the smallest AICc, and other models which have ΔAICc <2. (PDF) [file pone.0135585.s001.pdf]

	Estimate	p-v
(Intercept)	-0.8448	0.4735
**Extrinsinc factors**		
Mean human footprint	0.0564	0.0003
**Intrinsic Factors**		
Ecology		
Annual range of precipitations	-0.0002	0.0003
Mean temperature range	-0.0054	0.0309
Frugivory	0.0151	0.0058
Home range (log)	0.2171	0.0460
Life history		
Gestation	0.0138	0.0286
Female body mass (res)	0.3872	0.0761
Behaviour		
Mean group size (log)	-0.4166	0.0519
Socio-reproductive system:		
polygynous and promiscuous	-0.5674	0.2450
polygynous	0.4693	0.4014
promiscuous	0.1023	0.8362
solitary	-0.2164	0.7688

AICc = 288.20

	Estimate	p-v
(Intercept)	-0.8076	0.4919
**Extrinsinc factors**		
Mean human footprint	0.0580	0.0003
**Intrinsic Factors**		
Ecology		
Annual range of precipitations	-0.0002	0.0025
Mean temperature range	-0.0048	0.0569
Frugivory	0.0158	0.0050
Home range (log)	0.2963	0.0035
Life history		
Gestation	0.0096	0.1040
Behaviour		
Mean group size (log)	-0.3773	0.0377

AICc = 288.93

	Estimate	p-v
(Intercept)	0.4240	0.6414
**Extrinsinc factors**		
Mean human footprint	0.0586	0.0003
**Intrinsic Factors**		
Ecology		

Annual range of precipitations	-0.0002	0.0019
Mean temperature range	-0.0051	0.0446
Frugivory	0.0156	0.0057
Home range (log)	0.3407	0.0006
Behaviour		
Mean group size (log)	-0.3548	0.0519

AICc = 289.44

	Estimate	p-v
(Intercept)	-0.1091	0.3593
**Extrinsinc factors**		
Mean human footprint	0.0560	0.0005
**Intrinsic Factors**		
Ecology		
Annual range of precipitations	-0.0002	0.0019
Mean temperature range	-0.0048	0.0542
Frugivory	0.0157	0.0058
Home range (log)	0.2171	0.0050
Life history		
Gestation	0.0132	0.0426
Female body mass (res)	0.2890	0.1863
Behaviour		
Mean group size (log)	-0.3540	0.0510

AICc = 289.44

	Estimate	p-v
(Intercept)	-0.9326	0.4301
**Extrinsinc factors**		
Mean human footprint	0.0557	0.0004
**Intrinsic Factors**		
Ecology		
Annual range of precipitations	-0.0002	0.0004
Mean temperature range	-0.0057	0.0241
Frugivory	0.0150	0.0059
Home range (log)	0.2394	0.0315
Life history		
Gestation	0.0139	0.0284
Female body mass (res)	0.3212	0.1575
Behaviour		
Mean group size (log)	-0.4256	0.0474
Sexual dimorphism	-0.6112	0.3127
Socio-reproductive system:		
polygynous and promiscuous	-0.5291	0.2794
polygynous	0.5270	0.3487
promiscuous	0.1169	0.8132
solitary	-0.0829	0.9117

AICc = 289.77
